# Advancing the implementation of quality-assured oncological exercise therapy in Germany: protocol for the IMPLEMENT project

**DOI:** 10.1186/s12885-025-14064-5

**Published:** 2025-04-16

**Authors:** Mirko Brandes, Anika Berling-Ernst, Hansjoerg Baurecht, Wiebke Jensen, Miriam Götte, Anne Herrmann, Daniela Fuhr, Heike Schmidt, Antonia Lucas, Bernardine Madl, Simon Elmers, Thorsten Schmidt, Christine Welker, Andre Golla, Annalena Wehner, Dominik Morlok, Jana Rueter, Janina Meuer, Melanie Reitz, Jane Kersten, Rebecca Zimmer, Yvonne Gadczikowske, Renee Stark, Kathrin Hegenberg, Michael Laxy, Martin Halle, Patrick Jahn, Carsten Bokemeyer, Sebastian Theurich, Hajo Zeeb, Michael Leitzmann, Freerk T. Baumann, on behalf of the IMPLEMENT consortium

**Affiliations:** 1 Leibniz-Institute for Prevention Research and Epidemiology – BIPS, Bremen, Germany; 2 Department I of Internal Medicine, Center of Integrated Oncology Aachen Bonn Cologne Duesseldorf, University Hospital of Cologne, Cologne, Germany; 3 Department of Epidemiology and Preventive Medicine, University of Regensburg, University Hospital Regensburg, Regensburg, Germany; 4 Department of Oncology, BMT with Section Pneumology, Hubertus Wald Tumor Center - University Cancer Center Hamburg, University Medical Center Hamburg-Eppendorf, Hematology, Germany; 5 Department of Pediatric Hematology/Oncology, Pediatrics III, West German Cancer Center, University Hospital Essen, Essen, Germany; 6 Department of Epidemiology and Preventive Medicine, Medical Sociology, University of Regensburg, Regensburg, Germany; 7 Medical Faculty of Martin, University Clinic and Outpatient Clinic for Radiotherapy, Luther University Halle-Wittenberg, Halle, Germany; 8 University Cancer Center Schleswig-Holstein, University Hospital Schleswig-Holstein, Kiel, Germany; 9 Professorship of Public Health and Prevention, School of Medicine and Health, Technical University of Munich, Munich, Germany; 10 Department of Medicine III and Comprehensive Cancer Center (CCC Munich LMU), LMU University Hospital Munich, Munich, Germany; 11 School of Medicine and Health, Department for Preventive Sports Medicine and Sports Cardiology, Technical University of Munich, TUM University Hospital, Munich, Germany; 12 Health Sciences Bremen, University of Bremen, Bremen, Germany; 13 West German Cancer Center, University Hospital Essen, Essen, Germany; 14 Health Service Research Working Group| Acute Care, Medical Faculty of Martin Luther, University Medicine Halle (Saale), University Halle-Wittenberg, Halle, Germany; 15 Department of Internal Medicine III, University Hospital Regensburg, Regensburg, Germany; 16 Bavarian Center for Cancer Research (BZKF), LMU Munich, TU Munich, and Regensburg, Germany; 17 DZHK (German Center for Cardiovascular Research), partner site Munich Heart Alliance, Munich, Germany

**Keywords:** Oncological exercise therapy, Implementation, Cancer, Strategies

## Abstract

**Background:**

Although quality-assured oncological exercise therapy (qOET) has proven effective for cancer patients at any stage of treatment and during aftercare, it is not yet incorporated into standard care in Germany and, to the best of our knowledge, in any other country. A collaboration involving eight German research institutions was initiated to investigate the barriers and facilitators to implementation and promote the wider dissemination of qOET for cancer patients across various settings in Germany.

**Methods:**

The IMPLEMENT project is designed as an exploratory study with a quasi-experimental design and a mixed-methods approach, combining qualitative and quantitative data collection. Institutions involved in the treatment and/or aftercare of cancer patients will be approached to identify key barriers and facilitators of qOET. Based on these findings, a set of implementation strategies (IPS) will be developed, implemented, and evaluated to facilitate the delivery of qOET for cancer patients. We aim to develop a variety of IPS for different contexts: urban settings (e.g. qualifying local aftercare institutions to provide qOET); rural settings (e.g. a hybrid approach for areas with limited access to local qOET services); adult cancer patients (e.g. focussing on patient education); and children and young cancer patients (e.g. offering consultations with training therapy experts). Additionally, interface management, training concepts, digital support, and economic evaluation will be considered to further promote the wider dissemination of qOET. The impact of the IPS will primarily be measured by the reach of qOET, assessed by comparing the number of cancer patients receiving qOET before and after implementation.

**Discussion:**

The aim of IMPLEMENT is to address key barriers and facilitators for the implementation of qOET in Germany, and to increase the number of cancer patients receiving qOET in the long term. Following the project, successful IPS will be disseminated for broader application. The IMPLEMENT consortium aims to make a significant contribution to the long-term integration of qOET into the standard care of cancer patients in Germany and prospectively for other countries as well.

**Trial registration:**

Clin. Trials: NCT06496711. German Clinical Trial Register (Deutsches Register Klinischer Studien - DRKS): 00032292. Bavarian Cancer Research Center (Bayerisches Krebsforschungszentrum (BZKF bzw. ZKS): DZ-2024-2165-9

**Supplementary Information:**

The online version contains supplementary material available at 10.1186/s12885-025-14064-5.

## Introduction

In 2020, 231,400 women and 261,800 men in Germany were newly diagnosed with cancer, corresponding to incidence rates of 549 and 638 new cases per 100,000 inhabitants, respectively [1]. The 5-year absolute survival rates were 58% for women and 52% for men. Among children aged under 18 years, 2,250 new cases were reported, with incidence rates of 16.1 for girls and 18.9 for boys per 100,000 children and a 5-year survival rate of approximately 88% [1]. In terms of population burden, cancer is amongst the top five causes of death in high-income countries, including Germany [1, 2]. For Germany, the incidence of cancer is projected to increase by 23% by 2030 due to an ageing population and factors such as environmental and lifestyle changes [1]. Consequently, the prevention and treatment of cancer remains a major challenge for the German healthcare system.

Supplementing standard cancer treatment with exercise therapy has been proven highly effective in improving patient related outcomes. Meta-analyses of individual patient data and systematic reviews demonstrate the beneficial effects of physical exercise on quality of life, clinical outcomes, and physical functioning in cancer patients. These findings support the recommendation of exercise therapy irrespective of cancer type [3, 4]. Moreover, meeting physical activity recommendations is strongly associated with both the prevention and improved survival rates for several cancer types [5]. Considering its impact on treatment-related adverse effects and side effects, such as fatigue, depression, sleep disturbances, and bone health, exercise is recommended across the entire cancer care continuum [6, 7]. It should follow established exercise guidelines proposed for cancer patients [8]. To ensure high-quality and safe exercise therapy for cancer patients, a personalised concept of quality-assured oncological exercise therapy (qOET), based on oncological training therapy (OTT), has been proposed for implementation in Germany [9]. An adapted concept for promoting physical activity and exercise therapy has been established for paediatric cancer patients as well as adolescents and young adults (AYAs) [10] and is outlined in evidence-based guidelines for Germany [11]. A detailed overview of the principles of qOET for adults and paediatric cancer patients is given in supplemental file 1. Briefly, qOET is characterised by all of the following key requirements:

- Therapists are trained or hold a degree or professional training in physiotherapy, movement and exercise science, or a related field.
- Therapists have completed professional training in OTT or an equivalent programme designed for cancer patients.
- qOET is tailored to individual patient needs, symptoms, cancer types, and therapy-related side effects.
- qOET delivery is developed collaboratively in dialogue with the cancer patient.
- Training facilities and equipment meet hygiene, maintenance, and safety standards.
- Cancer patients undergo physical safety checks by clinicians prior to qOET to ensure no contraindications exist.
- qOET is supervised by skilled therapists, maintaining a maximum therapist-to-patient ratio of 1:5 for adult patients, 1:2 for paediatric cancer patients in acute therapy, and 1:7 in follow-up care.

Despite compelling strong evidence supporting the benefits of exercise therapy for cancer patients, neither exercise therapy nor qOET is currently integrated into the routine treatment of cancer patients in Germany [12]. This is attributed to multiple implementation barriers, such as organisational challenges, lack of supportive structures, insufficient standardised training, fragmented care between hospitals and practices, and unclear reimbursement processes [13]. A questionnaire-based assessment from 2019 revealed that only 22.7% of surveyed cancer centres met the minimum requirements for qOET, and merely 30% of cancer patients participate in any form of exercise therapy during treatment [14]. Moreover, the availability of exercise therapy in Germany is highly inconsistent. Larger comprehensive cancer centres (CCCs), primarily linked to university hospitals in major cities, tend to offer qOET to cancer patients, while opportunities for exercise therapy in rural areas remain scarce. Additionally, while some private rehabilitation facilities offer exercise therapy, it is often unclear whether these facilities provide professional-level care or adhere to qOET guidelines. In the paediatric setting, the national German Network ActiveOncoKids (NAOK) supports patients in identifying personalised exercise options [15] and assists clinics and centres in developing and implementing exercise programmes [16]. According to the 2024 NAOK database, 36 out of 60 acute paediatric clinics in Germany are part of the NAOK, with 33 (55%) currently offering exercise therapy to varying extent.

With respect to the low number of centres offering qOET and high levels of physical inactivity in adult and paediatric cancer patients, the fragmented landscape highlights the urgent need for national efforts to integrate qOET into routine cancer care in Germany [7, 17]. The evidence supporting qOET for cancer patients, combined with the current lack of its implementation in Germany, forms the overarching rationale for the IMPLEMENT project. IMPLEMENT aims to strengthen the implementation of qOET in cancer patients across Germany by identifying and addressing multiple barriers and facilitators. The project aims are structured around the RE-AIM (Reach, Effectiveness, Adoption, Implementation and Maintenance) framework [18]. The primary outcome of IMPLEMENT is to expand the reach of qOET, specifically by increasing the overall number of cancer patients receiving qOET. Additionally, secondary outcomes have been developed in line with the RE-AIM framework (see additional file 2 for the full list). Key secondary outcomes involve:

- Increasing the number of cancer patients reporting a significant positive impact of qOET on their daily life (effectiveness).
- Expanding the number of institutions offering qOET (adoption).
- Promoting the qOET professional training programme and increase certification by at least 50% of therapists (implementation consistency).
- Ensuring the sustainability of implementation options to provide qOET provision beyond the three-year project period through the promotion of funding opportunities (maintenance).

## Methods

### Design of the project

The IMPLEMENT project is designed as an explorative study with a quasi-experimental design using mixed-methods. The consortium consists of researchers and clinicians from eight university hospitals and research institutions across Germany (the “IMPLEMENT sites”, further details at https://cio.uk-koeln.de/leben-mit-krebs/bewegung/studien-und-publikationen/implement-projekt/). To enhance the implementation of qOET in Germany, IMPLEMENT’s core objective is to iteratively develop, implement, and evaluate various implementation strategies (IPS) over a three-year period. This process involves three key steps. Step 1 focuses on identifying key barriers and facilitators for implementing qOET in various settings, which is conducted at the project’s outset to inform the development of IPS. Step 2 involves the iterative design, implementation, and evaluation of these IPS throughout the three-year period. The IPS are designed by the IMPLEMENT sites (see below for further details) and implemented in the “network” of each IMPLEMENT site. The “networks” of each single IMPLEMENT site potentially include any collaborating institutions and/or stakeholders involved in the treatment of cancer patients, such as clinics, physicians, rehabilitation centres, or physiotherapists. An expansion of the networks is a desired consequence of steps 1 and 2. Finally, step 3 encompasses a comprehensive analysis of qOET implementation in Germany and a process evaluation of the IPS before the end of the funding period. In this step, promising IPS will be disseminated widely to further facilitate the adoption of exercise therapy among cancer patients.

**Fig. 1:**
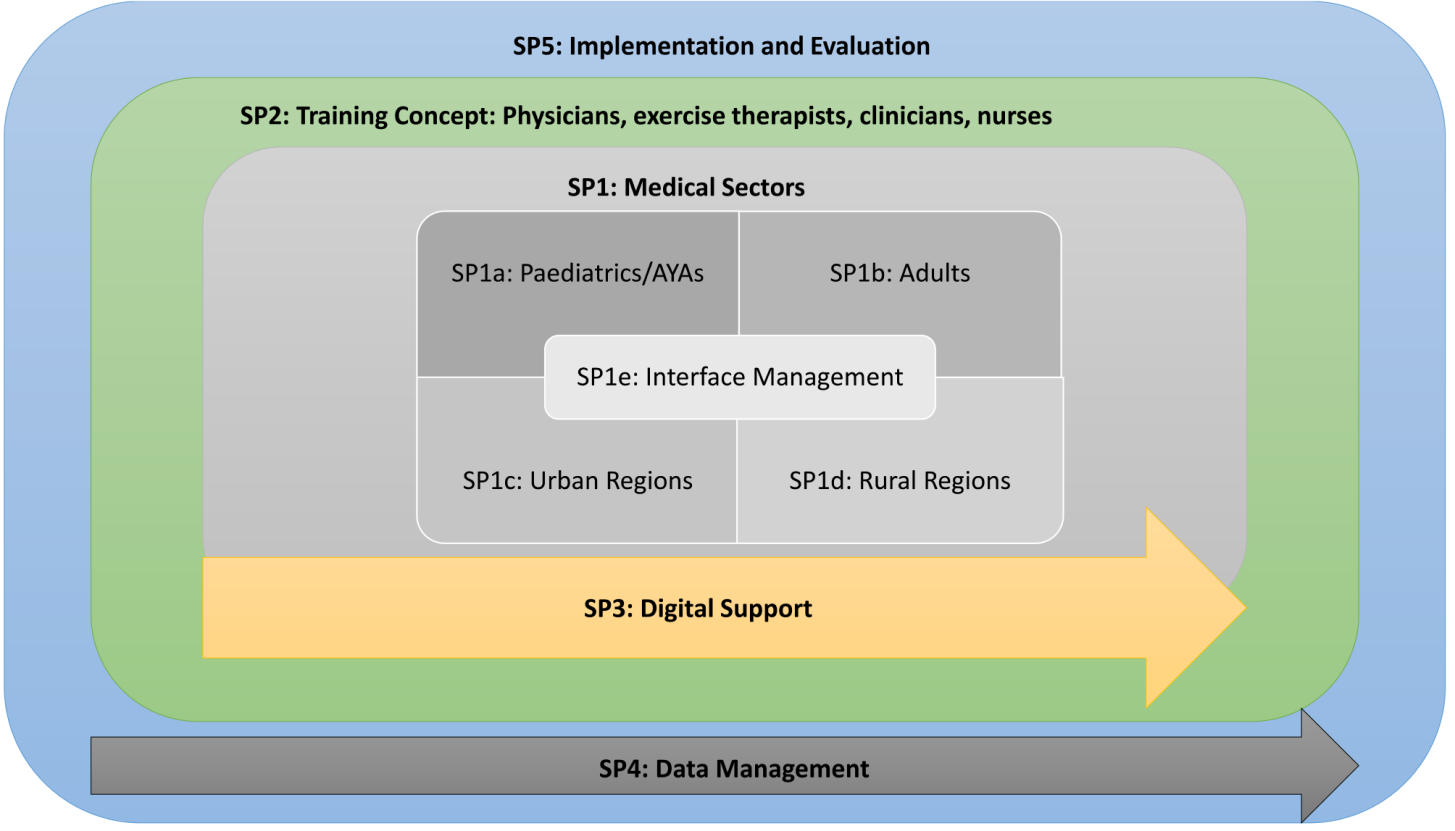
Structure of the overall IMPLEMENT project and subprojects (SP). AYAs: adolescents and young adults, aged 18–39 years. Adults: aged 40 + years

The IMPLEMENT project is divided into five partially overlapping subprojects, each targeting a distinct population or fulfilling a specific role within the overall framework of the project (Fig. 1).

Subproject1 (SP1) covers all relevant care sectors where qOET can be applied. It is further subdivided into the following components:

- SP1a, addressing paediatric and AYA cancer patients.
- SP1b, addressing adult cancer patients.
- SP1c, addressing cancer patients living in urban areas.
- SP1d, addressing cancer patients living in rural areas.
- SP1e, addressing interfaces and overlaps among SP1a-d.

**Fig. 2:**
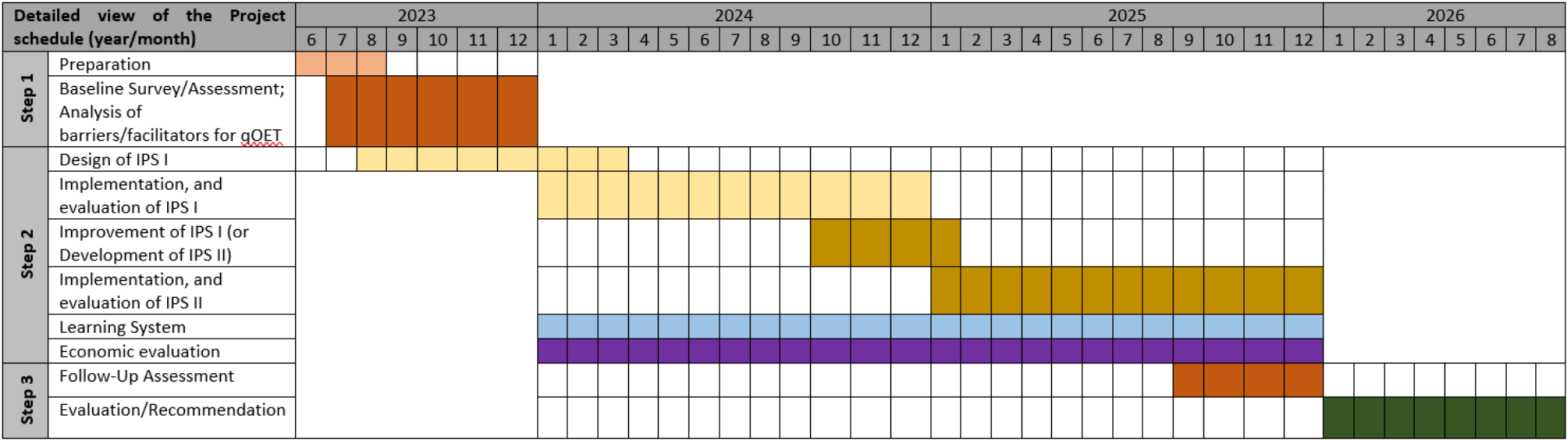
Overall schedule of the IMPLEMENT project. IPS = implementation strategies; qOET = quality-assured oncological exercise therapy

Subprojects of SP1 as well as the overlapping SP2 (focussing on the education and training of physicians, exercise therapists, clinicians, nurses) are designed to develop, implement, and evaluate IPS to achieve the primary and secondary aims of IMPLEMENT. These IPS include standardised operating procedures (SOPs) with indicators that can be used for process evaluations, to enable other IMPLEMENT sites to run the IPS in their network. SOPs are presented and discussed within a *“Learning System”*, a bi-weekly online meeting of all IMPLEMENT sites (details see below). Once developed, all IMPLEMENT sites are encouraged to implement all IPS that fit to the needs of their networks in the implementation phase, which covers two years of the IMPLEMENT project (see Fig. 2). Subprojects are also responsible for monitoring and evaluating the process of their IPS throughout the implementation phase. SP3 aims to develop digital support for SP1 and SP2, such as web-based resources and social media content. SP4 ensures secure data management structures for all data collected within IMPLEMENT, including the establishment of a REDCap-based database. SP5 is designed to evaluate the overall outcome of IMPLEMENT and to advise on generic IPS that can be utilised in SP1 and SP2.

Ethics approval for IMPLEMENT was initially obtained from the University of Bremen, Germany (ref. 2023-16). All participating institutions received ethics approval from their respective ethics committees based on this initial authorization.

### Learning System

To continuously monitor and modify IPS throughout the implementation phase, a so-called *“Learning System”* will be established. The core of the *Learning System* is a bi-weekly, three-hour online meeting, where each subproject reports the progress and/or new developments of each IPS. This is followed by reports from all IMPLEMENT sites currently implementing specific IPS. Thus, findings with respect to implementation issues raised in one region in Germany are openly shared with the entire IMPLEMENT consortium, allowing for the discussion of consequences and potential solutions applicable across all regions. If necessary, minor changes to the IPS SOPs can be agreed upon within each *Learning System* session. Extensive adjustments or changes must obtain approval from the primary investigators. Other issues, such as mandatory changes to the documentation process, can be raised during *Learning System* sessions. These are then delegated to specialised online meetings with expert sub-groups.

## Step 1: analysis of barriers and facilitators for qOET

In step 1, we will map, expand and inform the networks and conduct a needs assessment to evaluate the requirements for implementing qOET in participating institutions. The IMPLEMENT sites will collect relevant information on institutions in their network as well as potential new institutions, including key characteristics and contact opportunities. Connections will be established with these institutions and key stakeholders to recruit participants for the needs assessment and to develop and adapt the IPS according to the specific context.

To explore determinants of implementation, we employ the Consolidated Framework for Implementation Research (CFIR [19, 20]). Briefly, the CFIR proposes exploring the relevance of 48 influencing factors (‘constructs’) across five domains (outer setting, inner setting, individuals, implementation process, and innovation), each related to the implementation process. To streamline the process, an IMPLEMENT expert panel distils the CFIR constructs to those most critical for the implementation of qOET in Germany. A questionnaire based on these prioritised constructs will then be distributed across the networks. Based on the results, we develop topic guides for qualitative interviews with local stakeholders and cancer patients. Additionally, a quantitative, questionnaire-based assessment of qOET with key stakeholders at regional or supra-regional level will support the analysis of barriers and facilitators. A deductive analysis of the interviews, alongside insights derived from the quantitative questionnaires, will identify the most critical barriers and facilitators for qOET implementation and inform the development of IPS.

## Step 2: design, implementation, and evaluation of IPS for medical sectors and training

### SP1a: Paediatrics and AYAs

The aim of SP1a is to implement qOET in paediatric oncology clinics at IMPLEMENT sites and affiliated institutions, thereby increasing the number of children, adolescents and young adults with cancer participating in qOET. Two core aspects are pursued to achieve this goal: [1] the development and expansion of local qOET programmes and [2] the integration of staff and patients into the national structures of the Network ActiveOncoKids (NAOK).

Based on a baseline assessment, clinic-specific barriers and facilitators are identified, and the paediatric oncology clinic is classified on a 5-level scale (ranging from Level 1: No physical activity offers available, to Level 5: qOET is reimbursable through statutory health insurance and available across all treatment phases). IPS are tailored to the respective level and existing barriers are carried out in close collaboration with the NAOK.

Adolescents and young adults (15–39 years) are currently treated at these sites under differing jurisdictions, often without qOET programmes tailored to their specific needs. Therefore, an inter-site consultation service will be established to address this gap.

### SP1b: adults

The goal of SP1b is to establish and optimize qOET for adults, primarily educating patients and physicians. The aim of the SP is to empower cancer patients to take an active role in their care, fostering patient empowerment, and guiding them toward suitable qOET programmes. To achieve these goals, SP1b is developing a range of information materials, including flyers, slides and inserts. These will provide cancer patients with information from diagnosis through to aftercare, aiming to encourage them to consult their doctors about qOET and inquire with their health insurance companies regarding coverage. Each informational material will feature a QR code that directs cancer patients to the digital information platform developed by SP3, where they can access further information on qOET for cancer. The platform also includes a search tool that enables cancer patients to locate local care facilities offering qOET services. Additionally, SP1b is developing an online consultation service for cancer patients interested in qOET. This consultation, estimated to last 20–30 min, is designed to provide cancer patients with a comprehensive understanding of the effectiveness and importance of qOET, fostering a positive attitude and motivation toward participation. The objective is to enhance the number of patients who are informed about and engage with qOET.

### SP1c: urban areas

The main goal of SP1c is to increase the number of local institutions, particularly physical therapy facilities, capable of providing qOET to cancer patients. In a first step, local physical therapy facilities are identified through personal contacts and/or a web-based search. If available, the facility’s website is reviewed for information regarding services for cancer patients and relevant billing options. Secondly, information materials outlining the benefits of qOET, current training and education options for health care professionals (see SP2), and details about the IMPLEMENT project are sent to the facility by mail and email. With the facility’s consent, IMPLEMENT staff conduct a personal visit to evaluate which qOET requirements, relating to knowledge, equipment, spatial requirements, staff qualification, and financing, are not being met. Finally, facilities receive tailored support to address the identified gaps until all qOET requirements are fulfilled. If staff qualification is identified as a need, therapists are referred to SP2 for appropriate training. Institutions fulfilling all qOET requirements are encouraged to participate in the referral management processes established under SP1e.

### SP1d: rural areas

The subproject focuses on the provision of qOETs in rural areas, which are particularly characterised by limited infrastructure and challenging accessibility. To address barriers such as restricted services and long travel distances, a hybrid qOET service model incorporating digital technologies will be developed. A co-creative approach will ensure that the needs of both cancer patients and service providers regarding digital components are adequately considered. The hybrid qOET service will be progressively implemented across several institutions and evaluated from multiple perspectives, including user satisfaction, practical feasibility, and overall effectiveness.

### SP1e: interface management

SP1e activity aims to enhance collaboration across acute care, rehabilitation, and aftercare sectors. Mixed-methods analyses will identify barriers to access and service integration, guiding the development of interface tools and transition protocols to strengthen sectoral links. As all IPS address different stakeholders along the patient journey, the SP1e focuses on interconnecting stakeholders. As part of a digital referral management and reminder system, information about facilities offering qOET is passed on to both doctors in clinics and to practitioners. This enables health personnel to make specific recommendations for cancer patients.

### SP2: professional training and education strategy

SP2 aims to improve qOET delivery through evidence-based training for all health care professionals involved in cancer care. Based on OTT guidelines (60 units), physiotherapists and exercise therapists can either attend an established five-day professional training programme or participate in a newly developed OTT online education model, which consists of an e-learning platform combined with online-in-person sessions. These programmes focus on addressing the specific needs of cancer patients and structuring qOET exercises effectively. Shorter training sessions (90 min) are available for other health professionals, such as nurses, medical assistants, clinicians, nutritionists, and psycho-oncologists. These sessions prepare participants to act as “exercise therapy navigators”, responsible for identifying, informing, and referring suitable cancer patients to qOET programmes.

### Supporting subprojects for the design, implementation and evaluation of the IPS

#### SP3: digital support

SP3 focuses on developing a digital information platform (DIP) at www.bewegung-bei-krebs.org. The platform provides easy access to information about qOET services for patients, their families, and other interested individuals through various media formats, including text, videos, and e-learning tools. It also features an interactive tool to assist users in locating the nearest qOET providers. In addition, the platform offers training materials and re-imbursement advice for therapists and healthcare staff.

Newsletters and event announcements are made available to enhance understanding and awareness of qOET delivery. Additionally, secure resources for the IMPLEMENT consortium, such as protocols, SOPs, and data management tools, are provided to support the effective implementation and management of the project.

#### SP4: data management

SP4 establishes a secure, standardised data management system that ensures efficient organisation, safe storage, and compliance with data protection regulations. Key tasks include integrating data from all sites, mainly using the web-based electronic data capture system REDCap [21, 22], conducting continuous quality control (including standardisation, validity, and reliability), and providing cleaned datasets for statistical analysis, while maintaining the independence of data management and evaluation. Data management plays a crucial role in monitoring the progress of IPS, such as tracking the number of sites where IPS are currently being implemented, as well as facilitating process evaluations. All procedures follow GDPR regulations and adhere to the TMF e.V. – Technology and Methods Platform for Networked Medical Research (German non-profit organization) principles for medical research, ensuring confidentiality and data integrity within the EU.

#### SP5: implementation and evaluation

SP5 provides guidance on IPS development, rooted in implementation theory, and evaluates the overall outcomes of the IMPLEMENT project. Both dedicated and generic models and frameworks of implementation theory are provided and tailored to each IPS in close collaboration with the relevant subproject. This process also includes a dedicated process evaluation for each IPS to assess its implementation effectiveness. Detailed information on the overall evaluation of IMPLEMENT is provided below (see step 3).

#### Additional project component: economic evaluation

This involves developing a cost reimbursement model for qOET by analysing current billing structures, resource requirements, and service scalability. Further, information derived from the questionnaire for cancer patients and stakeholders (see step 3), such as the number of patients unwilling to utilize qOET, is integrated in the economic evaluation. The aim is to establish sustainable financial models for integrating qOET into routine care.

## Step 3: overall evaluation of IMPLEMENT

The assessment of the outcome of IMPLEMENT is primarily guided by the RE-AIM framework. The evaluation builds on the qualitative and quantitative data collected at both baseline and follow-up:

- An assessment of RE-AIM categories [18] using a questionnaire completed jointly by the addressee and a researcher for all institutions providing qOET.
- An individual questionnaire for cancer patients and stakeholders.
- Qualitative interviews of cancer patients and stakeholders.

Baseline assessments take place prior to the delivery of any initial IPS (November 2023 – February 2024). The follow-up assessment is scheduled for November 2025 – February 2026. Newly participating institutions joining the IMPLEMENT network and those already providing qOET at the time of recruitment will complete the baseline assessment as soon as possible after joining.

### Assessment of RE-AIM

All institutions within the IMPLEMENT network involved in the delivery of qOET are assessed using a dedicated questionnaire (see Supplemental file 3 for full questionnaire). This tool collects relevant data to estimate the impact of the IMPLEMENT project with respect to the primary and secondary outcomes (see add file 2):

- The RE-AIM categories of Reach, Effectiveness, Adoption, Implementation and Maintenance.
- Indicators of qOET to ensure that all conditions for providing a qOET are met by the institution.

Each institution is required to report the number of cancer patients who received qOET during a specified three-month period in the previous year, as well as the overall number of cancer patients with a primary diagnosis over the preceding year. Assuming the overall number of cancer patients remains relatively stable from year to year, we aim to calculate the proportion of cancer patients receiving qOET. We anticipate that this proportion will increase due to the specific IPS interventions.

### Questionnaire for cancer patients and stakeholders

In addition to qualitative interviews, focus groups, and the CFIR-based questionnaire, cancer patients and stakeholders at the IMPLEMENT sites (e.g., clinicians, nurses, and other health care professionals) are asked about barriers and facilitators to implement qOET. This is conducted using a self-developed, literature-based questionnaire completed either online or via pen-and-paper.

### Interviews of cancer patients and stakeholders

Semi-structured interviews and focus groups with cancer patients and stakeholders are conducted cross-sectionally to gain an in-depth understanding of their views, experiences, and expectations related to qOET. The interview and focus group guides are designed to explore facilitators and barriers to participation in qOET, including access to information and referral processes. In addition, participants are asked how qOET could be improved, for example in terms of accessibility and feasibility. The development of these literature-based guides is informed by the RE-AIM framework as well as preliminary findings from the baseline CFIR analysis. Thus, these interviews and focus groups also help to identify whether there is a need to adapt and refine the implementation strategies, particularly in relation to the RE-AIM category of “maintenance”. Purposive sampling will be employed to recruit participants from diverse regions, encompassing various socio-demographic and disease-related characteristics (for patients) or professional backgrounds (for stakeholders). This approach will help identify potential differences between urban and rural settings, as well as individual variations.

### Study population

The design of the IMPLEMENT project necessitates data collection involving various cancer patient populations and stakeholders. This includes stakeholders and cancer patients from all IMPLEMENT sites and their networks who may potentially deliver qOET, refer cancer patients to qOET, or provide related advice. This also includes associations, private medical practitioners, and physiotherapy facilities, and cancer patients eligible to participate in qOET (i.e., without contraindications for qOET). Clinics and institutions will provide aggregated information regarding their relevant patient groups.

### Recruitment

#### Recruitment of collaborating institutions

With respect to institutions, the IMPLEMENT sites have established broad networks of cooperating institutions, including medical practice, hospitals, counselling centres, physiotherapy practices, and sports, exercise, and rehabilitation facilities. IMPLEMENT sites also invite other institutions potentially interested in introducing qOET through various channels, including print and digital media, as well as social media platforms. Inclusion criteria for collaborating institutions require involvement of counselling and/or treating cancer patients, including aftercare. Institutions unwilling to offer any kind of exercise therapy aligned with qOET are excluded. In such cases, institutions are invited to take part in an interview to explore the reasons behind their reluctance to implement qOET. Only those institutions that also decline to participate in the interview are fully excluded from the IMPLEMENT project.

#### Recruitment of cancer patients

IMPLEMENT sites employ all available strategies to recruit cancer patients and stakeholders. Our project aims for a total sample size of at least 600 cancer patients, with 300 patients per time point, to ensure adequate data for analysis. Although no formal sample size calculations were performed, due to the absence of prior research on which to base assumptions and the quasi-experimental study design, this sample size is considered sufficient to effectively address the research question. For example, at an alpha level of 0.05, our sample size estimates suggest the ability to detect a moderate effect size of 0.3 on a four-point Likert scale, assessing agreement and disagreement with barriers or facilitators, with a power of 95%. All cancer patients (across all entities) are eligible for inclusion. Cancer patients are excluded if mental and/or physical limitations prevent participation in qOET, such as unstable heart rhythm disorders.

#### Serious adverse events

Due to the nature of the IMPLEMENT project, no interventions are made in the current treatment of cancer patients. However, if the proportion of institutions providing qOET declines by 25% or more as a result of the project’s actions, the implementation of IPS at the affected institutions will be suspended to ensure that patient autonomy remains uncompromised.

#### Data management and processing

Study data are collected and managed using REDCap electronic data capture tools, operated by the University of Regensburg’s Institute of Epidemiology and Preventive Medicine (SP4). Data entry is web-based and automatically stored in a secure central study database with audit trails. The security of the REDCap implementation is guaranteed in particular by a three-server architecture, a firewall-protected subnet structure, exclusively encrypted data transmission (HTTPS/TLS), and daily backups. Access is secured through password protection and role-based permissions, granting data managers full control, while project staff can edit data only for their assigned participants.

### Data protection

The handling of data follows legal regulations (GDPR, national data protection laws) and is governed by a data protection plan. Data are collected and stored in anonymised or pseudonymised form. Personal data are collected only upon obtaining informed consent. Identifying data (e.g., contact details for longitudinal studies) are kept separate from pseudonymised study data; only pseudonymised data are transferred to the central study database. Audio and video recordings are transcribed and pseudonymised before being uploaded to the study database.

## Discussion

The IMPLEMENT project aims to achieve the ambitious goal of developing, implementing, and establishing a model access structure for quality-assured oncological exercise therapy in Germany within three years. This objective aligns with the principal goal of the German Cancer Aid, the project sponsor, to establish a sustainable, nationwide, and quality-assured exercise therapy infrastructure for cancer patients in Germany. The aim is to gather knowledge that has global significance, even if our health and social system differs from others. The initiative is being undertaken in collaboration with all relevant stakeholders. This discussion outlines the strengths of the approach, examines potential challenges and limitations, and identifies opportunities for optimisation.

Strengths and innovations of the IMPLEMENT project.

The methodological foundation in the RE-AIM framework [18] and the CFIR framework [19] is a standout feature of the IMPLEMENT project. These models facilitate comprehensive evaluations of reach, acceptance, implementation, effectiveness, and sustainability, while systematically addressing key implementation determinants. This approach supports not only the effective delivery of exercise therapy but also its long-term integration into routine care. Furthermore, alignment with evidence-based guidelines, such as the PRACTIS guide [23], provides a robust foundation for identifying and overcoming implementation barriers and facilitators.

Another key feature of the project is its mixed-methods approach, which enables an in-depth analysis of barriers and facilitating factors from both quantitative and qualitative perspectives. By integrating questionnaires, focus groups, and interviews, the project achieves a comprehensive understanding of the needs and challenges related to the implementation process. The active involvement of cancer patients and stakeholders in the need’s analysis and the participatory development of implementation tools fosters acceptance and enhances the practical relevance of the interventions.

Challenges and Limitations.

A primary limitation is the willingness of cancer patients to participate in exercise therapy and the readiness of physicians and nursing staff to support the interventions. These factors could impact on the speed and effectiveness of implementation. To address this, the project relies on early-phase analyses and continuous monitoring of potential barriers.

Another challenge is the heterogeneity of healthcare settings and services in Germany. Structural and personnel differences between urban and rural areas may impede the standardisation of interventions. To address this, IMPLEMENT will identify context-specific implementation approaches that are applicable in real-world settings, including those outside CCCs. Additionally, financial constraints could threaten the sustainability of the established structures. Nevertheless, planned discussions with health insurance providers to develop viable funding models represent a promising strategy for overcoming these obstacles.

Potential and Economic Perspectives.

The integration of an economic evaluation into the project is a significant strength. Analysing existing reimbursement options and developing a cost reimbursement model for exercise therapy provides a solid foundation for its inclusion in routine care. This is particularly relevant given the increasing importance of implementing cost-effective interventions within the healthcare system. In the long term, successful implementation could not only enhance the care of cancer patients but also serve as a model for other therapeutic approaches and healthcare systems.

Optimisation and Prospects.

The continuous integration of findings into a *Learning System* represents an innovative approach that enhances the project’s flexibility and ensures the quality and rigour of the implementation processes. Regular consortium meetings and the adaptation of interventions based on monitoring data are key components of this approach. Furthermore, increased incorporation of digital technologies, such as expansion of the digital information platform, could further enhance the reach and acceptance of the interventions.

In conclusion, the IMPLEMENT project is well-positioned to sustainably anchor high-quality exercise therapy in routine cancer care through its interdisciplinary and systematic approach. Recommendations for the implementation of qOET will be derived from the evaluation results and disseminated through scientific publications and relevant forums, such as networking conferences.

## Electronic supplementary material

Below is the link to the electronic supplementary material.

Supplementary Material 1

Supplementary Material 2

Supplementary Material 3

## Data Availability

No datasets were generated or analysed during the current study.

## Abbreviations

AYAs: Adolescents and young adults
BZKF: Bavarian Cancer Research Center (Bayerische Zentrum für Krebsforschung)
CCCs: Comprehensive cancer centres
CFIR: Consolidated Framework for Implementation Research
DIP: Digital information platform
DRKS: German Clinical Trial Register (Deutsches Register Klinischer Studien)
IPS: Implementation Strategie
NAOK: Network ActiveOncoKids
OTT: Oncological training therapy
qOET: Quality-assured, oncological exercise therapy
RE-AIM: Reach, Effectiveness, Adoption, Implementation and Maintenance
SOPs: Standardised operating procedures
SP: Subproject
TMF e.V.: Technology and Methods Platform for Networked Medical Research
