# Supplementary material for: Advancing the implementation of quality-assured oncological exercise therapy in Germany: protocol for the IMPLEMENT project
Source: BMC Cancer. 2025 Apr 16;25:710. doi: 10.1186/s12885-025-14064-5 (PMC12004655; doi:10.1186/s12885-025-14064-5)
Supplement: Supplementary file 1 — Supplementary Material 1 [file 12885_2025_14064_MOESM1_ESM.docx]

Additional file 1:

**Overview of Quality-Assured Oncological Sports and Exercise Therapy (qOET) for Adult and Paediatric Cancer Patients**

**Definition of qOET for Adult Cancer Patients**

- Quality-assured oncological sports and exercise therapy (qOET) is a personalized, evidence-based therapeutic approach designed for adult cancer patients. It aims to mitigate disease- and therapy-related effects and side effects across all treatment phases (acute, rehabilitation, aftercare, chronic). qOET integrates strength, endurance, coordination, and flexibility training in diverse settings, including clinical, private practice, home-based, and telemedical environments, ensuring adherence to biopsychosocial principles.
  For conducting qOET, sports-/exercise therapist or physiotherapist must hold a license of advanced training, specialized for cancer patients.
  Examples: Oncological training therapy (OTT) (1, 2) License “Oncology”, German Association for Health Sports and Sports Therapy e. V. (DVGS)

**Structural Requirements**:

- Facilities equipped for diverse training modalities, including strength (e.g., cable pull, theraband), endurance (e.g., treadmill, ergometer), and coordination training (e.g., sensorimotor tools).
- Adjustable equipment tailored to individual capacities and health documentation systems.
- Training programs monitored and controlled based on patient-specific risks and opportunities.

**Conceptual and Personnel Requirements**:

- Institutions must provide individualized assessment and follow-up systems.
- qOET must be supervised (presence/live-online) and should take place in small groups and depending on the setting and the individual situation of the cancer patient.

**Definition of qOET-P for Paediatric Cancer Patients**

Quality-assured oncological sports and exercise therapy in paediatrics (qOET-P) is a personalized movement-based care concept for children and adolescents with cancer. It aligns with current evidence (3) and the S2k guideline for pediatric oncology (4), promoting long-term health literacy and addressing biopsychosocial factors during all treatment phases. Delivered by qualified professionals, qOET-P supports supervised implementation in clinical, home-based, and telemedical settings.

**Examples**: Paediatric exercise therapy in paediatric oncology units and outpatient, during aftercare and survivorship, with appropriate qualifications, e.g. BOP (*Bewegungstherapeutische InterventiOnen in der Pädiatrischen Onkologie*).

**Structural Requirements**:

- Facilities must support movement promotion and therapeutic exercises with adaptable equipment for strength, endurance, coordination, and flexibility training.
- Clear documentation and communication systems regarding patients’ health status.

**Conceptual and Personnel Requirements**:

- Integration of qOET-P into hospital routines during acute therapy, with up to five sessions per week. Programs are voluntary, dialogue-oriented, and respect patient preferences.
- Implementation follows principles of individualization, pedagogical guidance, and scientific training practices.
- Supervision ratios vary: 1:1 or 1:2 in acute care and up to 1:7 in aftercare.
- Practitioners must have a professional background in sports/movement sciences or physiotherapy, with additional paediatric oncology training (ActiveOncoKids certification available from 2025) or relevant experience.

**Note**: Programs focused solely on skill development in specific sports are not classified as qOET-P.

References

1. Baumann FT, Hallek M, Meyer J, Galvao DA, Bloch W, Elter T. [Evidence and recommendations for oncologic clinical exercise - a personalized treatment concept for cancer patients]. Dtsch Med Wochenschr. 2015;140(19):1457-61.

2. Niels T, Schürhörster A, Wirtz P, Elter T, Baumann FT. Die Onkologische Trainings- und Bewegungstherapie (OTT) [Oncological training and exercise therapy (OTT)]. Bewegungstherapie und Gesundheitssport. 2018;34(01):50-4.

3. Gotte M, Gauss G, Dirksen U, Driever PH, Basu O, Baumann FT, et al. Multidisciplinary Network ActiveOncoKids guidelines for providing movement and exercise in pediatric oncology: Consensus-based recommendations. Pediatric blood & cancer. 2022;69(11):e29953.

4. Gesellschaft für Pädiatrische Onkologie und Hämatologie (GPOH), Arbeitsgemeinschaft Netzwerk ActiveOncoKids. S2k-Leitlinie Bewegungsförderung und Bewegungstherapie in der pädiatrischen Onkologie [S2k guideline on the promotion of physical activity and exercise therapy in pediatric oncology] 2021 [Available from: https://register.awmf.org/de/leitlinien/detail/025-036.
