# Supplementary material for: Advancing the implementation of quality-assured oncological exercise therapy in Germany: protocol for the IMPLEMENT project
Source: BMC Cancer. 2025 Apr 16;25:710. doi: 10.1186/s12885-025-14064-5 (PMC12004655; doi:10.1186/s12885-025-14064-5)
Supplement: Supplementary file 2 — Supplementary Material 2 [file 12885_2025_14064_MOESM2_ESM.docx]

**Please note that the following questionnaire is filled-in within a face-to-face online meeting**

**QUESTIONNAIRE**

**For institutions providing quality-assured sports and exercise therapy**

**Model project for the cross-sectoral implementation of a nationwide and quality-assured sports and exercise therapy for cancer patients**

Version 2.0, dated 12.12.2023

**Dear Sir/Madam,**

as part of the IMPLEMENT project, we are investigating strategies to enhance the dissemination of quality-assured sports and exercise therapy for oncological patients, ensuring that more patients can benefit from it. This questionnaire aims to capture potential effects of these strategies. We kindly ask you to complete the questionnaire, consisting of a maximum of 18 questions, to the best of your knowledge.

At certain points, you may not have the required information immediately available. In such cases, we ask you to obtain the necessary information from the relevant departments within your institution. You may revisit and update the questionnaire at any time. If possible, we kindly ask you to complete it within four weeks.

For any inquiries, you can reach us at: ---contact details---.

We appreciate your support!

**---contact details---**

You can also complete the questionnaire online.

Scan the adjacent QR code

OR visit: <https://umfrage.bewegung-bei-krebs.org/>
and enter the following access code: **---code---**

**Date of completion:**

__ __ Day __ __ Month __ __ __ __ Year

**General Information**

Firstly, we would like to gain an overview of how many patients receive quality-assured sports and exercise therapy in your institution annually.

Please note the different timeframes referenced in the questions.

1. Please specify the number of oncological patients (either in acute therapy or follow-up care) who participated in quality-assured sports and exercise therapy for the first time in your institution between 01.09.23 and 30.11.23. Each patient should only be counted once, regardless of how often they attended therapy during this period.

1.1. Can you differentiate between outpatient and inpatient oncological patients who participated in quality-assured sports and exercise therapy for the first time?

☐ Yes

☐ No

If **No**:

1.1.1. Total number of oncological patients who participated in quality-assured sports and exercise therapy for the first time between 01.09.23 and 30.11.23:

If **Yes**:

1.1.2. Number of **outpatient** oncological patients:

1.1.3. Number of **inpatient** oncological patients:

1.2. Can you differentiate between children, adolescents (under 18 years), adults (18 years and older), and AYAs (Adolescents and Young Adults, 15-39 years)?

☐ Yes

☐ No

If **Yes**:

1.2.1. Number of oncological patients from **paediatric oncology** (children and adolescents under 18 years):

1.2.2. Number of oncological patients categorized as **AYAs (15-39 years)**:

**Institutional Data**

2.1. Please specify the number of oncological patients who presented at your institution for the first time between **01.01.22 and 31.12.22**:

If **Yes** was selected in **1.2.**:

2.2. What percentage of oncological patients who presented at your institution for the first time in 01.01.22 – 31.12.22 belonged to **paediatric oncology** (children/adolescents under 18 years)?

If recordable:

2.3. What percentage of oncological patients who presented at your institution for the first time in 01.01.22 – 31.12.22 belonged to the **AYA** category (15-39 years)?

2.4. Additional information regarding data collection for 2.1. – 2.3. (e.g., data sources, estimates, etc.):

**Implementation of Quality-Assured Sports and Exercise Therapy**

1. Has quality-assured sports and exercise therapy for oncological patients been introduced in your institution within the past 12 months?

- Yes, on __ __ . __ __ . __ __ __ __
- No, it was introduced earlier

1. Is quality-assured sports and exercise therapy offered exclusively for certain oncological patient groups?

☐ Yes

☐ No, quality-assured sports and exercise therapy is available for all oncological patients

If **Yes**, specify the patient groups (such as “breast cancer”, PNP, paediatrics only, adults only,…):

1. Based on current staffing, spatial, and financial resources, how many oncological patients could your institution provide with quality-assured sports and exercise therapy **per week**? (Note: this does NOT refer to the total number of patients who are already training)
   ____________ patients per week
2. Is the quality-assured sports and exercise therapy individually tailored to patients’ needs and developed in consultation with them (and their guardians, if applicable)?

☐ Yes

☐ No

1. Are the symptoms and entities of oncological patients (e.g., PNP, fatigue) considered in quality-assured sports and exercise therapy?

☐ Yes

☐ No

1. Are the following **safety measures** ensured?

8.1. Are staff trained in hygiene regulations, safety, and occupational health measures, including handling training equipment?

☐ Yes

☐ No

8.2. Are training devices certified, maintained, and inspected for safe use?

☐ Yes

☐ No

8.3. Are the facilities (training rooms, changing rooms) and equipment hygienically safe (e.g., regular disinfection)?

☐ Yes

☐ No

**Medical and Supervision Aspects**

1. Are medical clearance tests carried out in your institution prior to the start of quality-assured sports and exercise therapy? (Multiple answers possible)

☐ All patients undergo a medical examination
☐ All patients undergo a stress ECG and echocardiogram
☐ All patients must provide a current medical report
☐ No, clearance is assumed if therapy is prescribed/referral-based

1. Is quality-assured sports and exercise therapy supervised by a therapist, being permanently present in the training room?

☐ No

☐ Yes, with an average ratio of **Therapists: Patients** → **____: ____**

1. How many therapists conducting quality-assured sports and exercise therapy hold additional qualifications for oncological patients (e.g., OTT, DVGS Oncology License)?

☐ All

**____%** of therapists

☐ None

1. Does your institution support further trainings/qualifications for therapists in oncology-specific sports therapy (e.g. OTT licence, DVGS oncology training licence)?

☐ Yes

☐ No

1. How many therapists attended a refresher or advanced training in oncology-specific sports therapy in the past 24 months?

☐**____** full-time staff

☐**____** part-time staff

1. What do you estimate what percentage of eligible oncological patients are informed about quality-assured sports and exercise therapy options? (Direct information such as personal discussions, patient brochures, etc.)

**____%** of patients

We appreciate your support!

For inquiries: ---contact details---
