# Supplementary material for: Advancing the implementation of quality-assured oncological exercise therapy in Germany: protocol for the IMPLEMENT project
Source: BMC Cancer. 2025 Apr 16;25:710. doi: 10.1186/s12885-025-14064-5 (PMC12004655; doi:10.1186/s12885-025-14064-5)
Supplement: Supplementary file 3 — Supplementary Material 3 [file 12885_2025_14064_MOESM3_ESM.docx]

**Additional file 2**

**Outcomes of the IMPLEMENT project**

As addressed in the core manuscript (see introduction; overall evaluation of IMPLEMENT (step 3)), the evaluation of the IMPLEMENT measures builds on the RE-AIM framework and is split in three assessment procedures:

1. An assessment of RE-AIM categories by questionnaire of all institutions which are providing qOET
2. A questionnaire for cancer patients and stakeholder
3. Interviews of patients and stakeholder

**Primary outcome**

RE-AIM category REACH

- Increase of the relative number of cancer patients provided with qOET, related to the overall number of cancer patients
  - Assessment method: Included in AM1. Institutions report cancer patients provided with qOET and overall number of cancer patients per year

**Secondary Outcomes**

*RE-AIM category ADOPTION*

- Increase of the overall number of institutions providing qOET to cancer patients
  - Assessment method: Included in AM1. Counting the number of institutions fulfilling the criteria for qOET and providing qOET to cancer patients
- Expansion of the cancer patient groups provided with qOET
  - Assessment method: Included in AM1. Institutions report the cancer patients groups provided with qOET (e.g. breast cancer, adults only,…)

*RE-AIM category EFFECTIVENESS (cancer patient level)*

- Proportion of cancer patients that report a positive impact of exercise therapy on their daily life
  - Assessment method: Included in AM2. Quality of daily life is assessed with the EORTC QLQ-C30 for adults and with the PedsQLTM4.0 for children and adolescents

*RE-AIM category IMPLEMENTATION*

- All IMPLEMENT sites and institutions a) promote the standardized qOET training program, hosted in Cologne, Germany, for adults, and in Essen, Germany, for pediatric patients, and b) 50% of exercise therapists of institutions providing qOET make use of the training programs.
  - Assessment method: Included in AM1. Institutions providing qOET report a) if they encourage their therapists to attend the training program, and b) the number of exercise therapist that took part in the training program within the last two years
- All institutions that provide qOET inform at least 80% of their patients about the local availability of qOET
  - Assessment method: Included in AM1. Institutions report the proportion of their cancer patients that are informed about qOET
- The qOET-supporting website developed by SP3 Digital Support is promoted to clinicians and cancer patients by IMPLEMENT sites and institutions
  - Assessment method: ?
- Collection of economic data to support the implementation of qOET
  - Assessment method: Availability of the economic evaluation report

*RE-AIM category MAINTENANCE*

- IMPLEMENT sites and institutions, which successfully implemented qOET during the project, will provide qOET to cancer patients beyond the project period
  - Assessment method: Included at AM1 (at follow-up only). Institutions are supposed to declare their intention to continue qOET.
- Information on financing models (e.g. with health insurances) from IMPLEMENT sites and institutions is summarized and is included in the recommendations for the sustainable implementation of qSBT
